# Supplementary material for: Diagnosis of Visceral Leishmaniasis in an Elimination Setting: A Validation Study of the Diagnostic Algorithm in India
Source: Diagnostics (Basel). 2022 Mar 9;12(3):670. doi: 10.3390/diagnostics12030670 (PMC8947297; doi:10.3390/diagnostics12030670)
Supplement: Supplementary file 1 [file diagnostics-12-00670-s001.zip › Figure S1.pdf]

Figure S1: Summary of the parasitemia among qPCR positive incident VL cases diagnosed at PHC level based on the diagnostic algorithm (n = 70). Parasitemia is presented on a logarithmic scale (log10(PGE/ml + 1)).

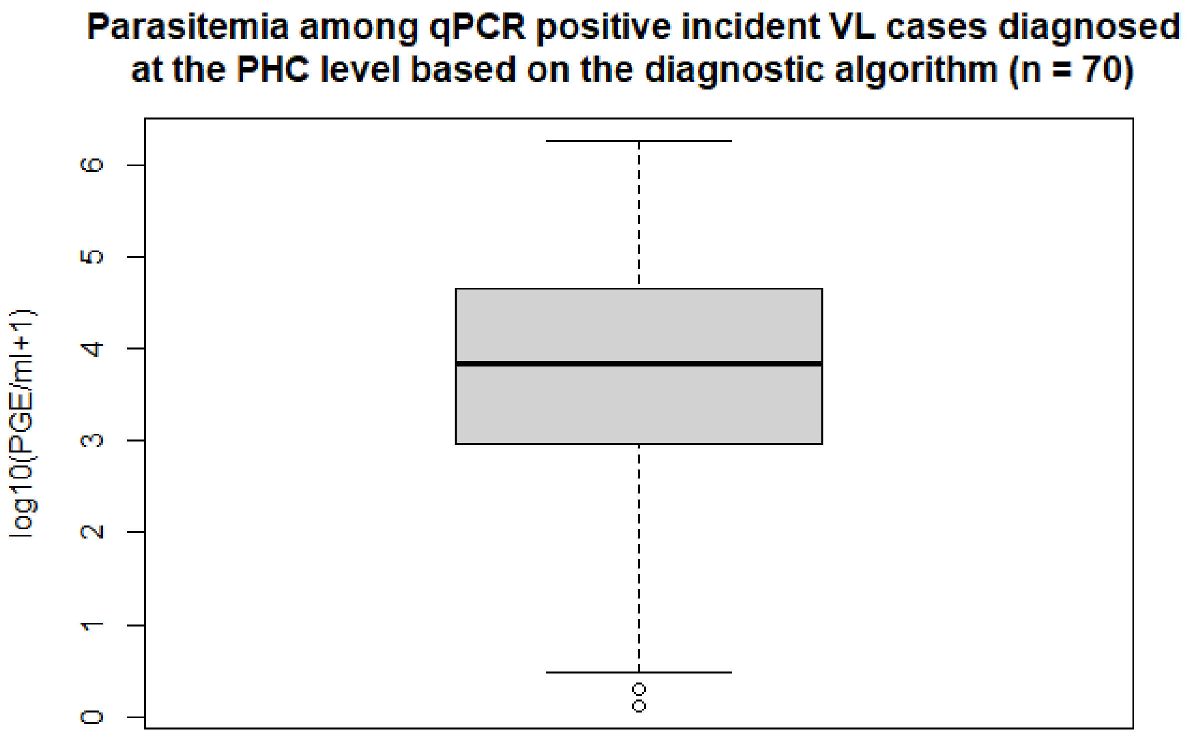

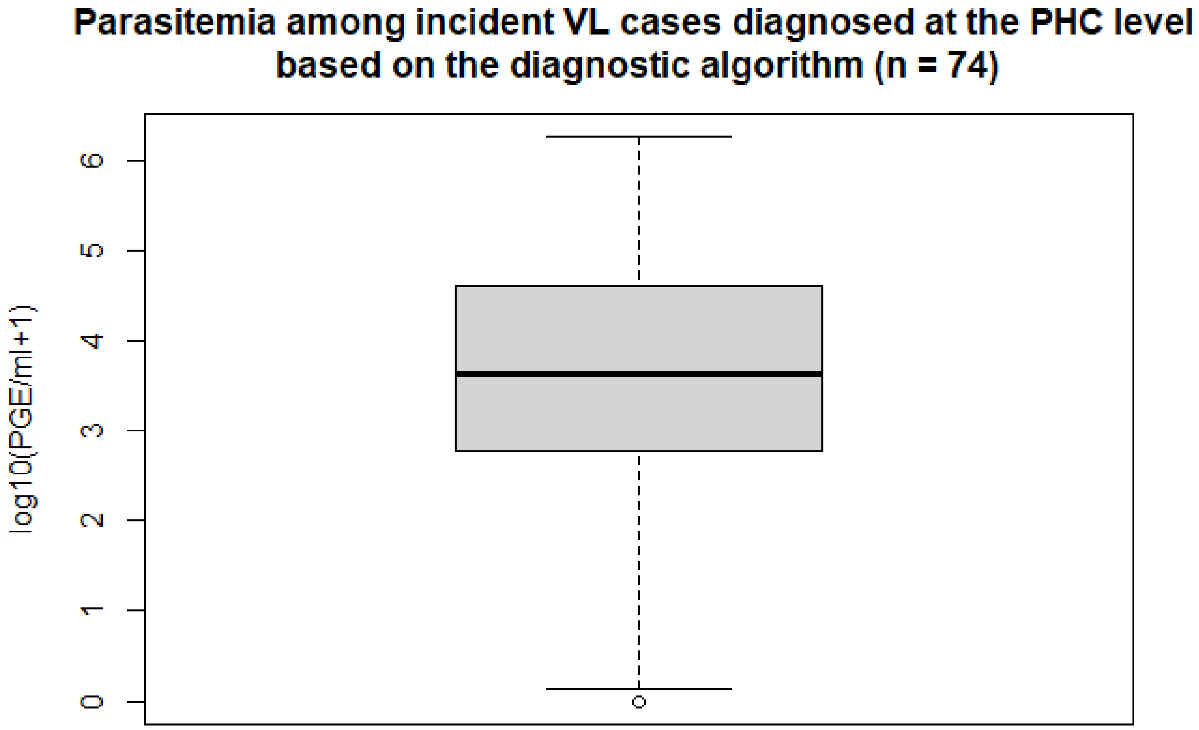
Parasitemia among incident VL cases diagnosed at the PHC level
based on the diagnostic algorithm (n = 74)
log10(PGE/ml+1)
0
1
2
3
4
5
6
