# Supplementary material for: Diagnosis of Visceral Leishmaniasis in an Elimination Setting: A Validation Study of the Diagnostic Algorithm in India
Source: Diagnostics (Basel). 2022 Mar 9;12(3):670. doi: 10.3390/diagnostics12030670 (PMC8947297; doi:10.3390/diagnostics12030670)
Supplement: Supplementary file 1 [file diagnostics-12-00670-s001.zip › Table S1.pdf]

Table S1: VL cases reported from the selected PHC involved in patient recruitment (2014 – 2018).

State	District	Block	2014	2015	2016	2017	2018	Grand Total	Population Block	Mean Annual Block incidence per 10,000 population (2014 - 2018)
Bihar	Saran	Garkha	106	81	109	110	120	526	268,156	3.92
Bihar	Saran	Dariapur	96	89	81	110	104	480	296,164	3.24
Bihar	Saran	Sonepur	35	35	36	92	96	294	232,340	2.53
Bihar	Muzaffarpur	Kanti	26	18	15	10	5	74	247,807	0.60
UP	Ballia	Bairia	1	1	3	2	14	21	176,824	0.24
UP	Ballia	Bansdih	0	0	0	1	1	2	162,127	0.02

PHC = Primary Health Care Center; UP = Uttar Pradesh. Population per block is based on the 2011 census data.
