# Supplementary material for: Diagnosis of Visceral Leishmaniasis in an Elimination Setting: A Validation Study of the Diagnostic Algorithm in India
Source: Diagnostics (Basel). 2022 Mar 9;12(3):670. doi: 10.3390/diagnostics12030670 (PMC8947297; doi:10.3390/diagnostics12030670)
Supplement: Supplementary file 1 [file diagnostics-12-00670-s001.zip › Table S2.pdf]

**Table S2:** VL cases reported from the selected clusters involved in recruitment of non-VL cases(2015 – 2020).

| State | District | Block | Cluster | 2015 | 2016 | 2017 | 2018 | Population cluster (at time of survey) | Status |
|---|---|---|---|---|---|---|---|---|---|
| Bihar | Saran | Dariapur | Rampur Jagdish | 0 | 4 | 9 | 3 | 4054 | CE |
| Bihar | Saran | Dariapur | Bishambarpur | 1 | 2 | 16 | 11 | 4024 | CE |
| Bihar | Muzaffarpur | Marwan | Jhakara Sekh | 0 | 0 | 0 | 0 | 3092 | PE |
| Bihar | Muzaffarpur | Marwan | Fanda | 0 | 0 | 0 | 0 | 3931 | PE |
| UP | Chandauli | Niyamatabad | Panndit K. N. | 0 | 0 | 0 | 0 | 954 | PE |
| Bihar | Muzaffarpur | Kanti | Panapur Kasba | 0 | 0 | 0 | 0 | 1043 | NE |
| UP | Varanasi | Kashi Vidyapeeth | Dangari Sarai | 0 | 0 | 0 | 0 | 4922 | NE |

*UP = Uttar Pradesh; Panndit K.N. = Panndit Kapurwa Nagar; CE = currently endemic cluster; PE = previously endemic cluster; NE = non-endemic cluster. Population cluster is based on the inhabitants of the respective clusters at the time of data collection (2019-2020).*
